# IDSov and the silent data revolution: Indigenous Peoples and the decentralized building blocks of web3

**DOI:** 10.3389/frma.2023.1160566

**Published:** 2023-05-19

**Authors:** Dillon Dobson, Adam Fernandez

**Affiliations:** Indigenous Peoples Law and Policy Program, University of Arizona, Tucson, AZ, United States

**Keywords:** Indigenous Data Sovereignty, Web3, international indigenous human rights, decentralized technologies, Native American

## Abstract

This article explores the technology underpinning the decentralized data revolution and encourages Indigenous Peoples (IPs) to secure their Indigenous Data Sovereignty (IDSov) over the Metaverse and Web3. More specifically, this article will survey blockchain technologies, exploring some disturbing colonial uses and providing an international legal framework that IPs can use to advance their IDSov internationally and domestically. This article will consider the role that cryptocurrencies, smart contracts, decentralized oracles, non-fungible tokens (NFTs), decentralized finance (DeFi), and decentralized autonomous organizations (DAOs) might play in advancing IDSov as it relates to western conceptualizations of Web3 and the Metaverse. The worldwide web's global data structure is undergoing a seismic shift that will significantly impact IPs. As inherent sovereigns, IPs are uniquely positioned to use and regulate these technologies in manners consistent with their cultural values and international indigenous human rights instruments. However, the march toward Web3 also looms menacingly over IPs. As such, we intend to examine IPs' novel risks and opportunities with Web3 and the Metaverse. We conclude by encouraging IPs to become fluent in the minutia of these technologies and to exert their inherent sovereignty over these nascent technologies in international and domestic arenas by building culturally informed systems to address their particularized needs. Future research should look toward the specific hurdles, and successes IPs are experiencing as they apply the technologies and principles discussed here.

## 1. Introduction

The world wide web is in the midst of a silent data revolution that will profoundly impact Indigenous Peoples (IPs) and Indigenous Data Sovereignty (IDSov). Lurking beneath the internet's relatively calm surface waters are the torrents of decentralization, steadily building pressure. Full of optimistic potential and dystopian horror, the new web has many faces and names—Web3, the Metaverse, Blockchain, and Cryptocurrency. Here, we will use the term Web3 as an umbrella term that includes and analyzes the Metaverse, Blockchain, and Cryptocurrency as constituent parts of the broader Web3 ecosystem. Web3 involves an evolution of western technologies and while we focus here primarily on North America IP implications and examples, our intent is to provide an overview of technological methodologies and legal analysis that are of value to IPs globally.

The internet is transitioning from a highly centralized and monopolistic amalgamation to something *different*. This new web purports to be more decentralized and democratic but there are many powerful and important critiques of the technologies and its harmful impacts Whether these optimistic visions will ultimately prevail remains unclear. It's still unclear what Web3 will look like and how it will function. Moreover, the particularized cultural and international conceptions and perceptions of Web3 are still taking shape. The contours of Web3's faces are still being outlined, enigmas of equally exciting and horrifying prospects. Enigmas that are well worth Indigenous curiosity, examination, and innovation.

This note aims to examine some of the constituent parts of Web2 and Web3, construct an international Indigenous human rights framework for the exertion of IDSov over Web3, and alert IPs to some of the alarming neocolonial Web3 efforts that might undermine their IDSov. Additionally, this note encourages IPs to begin the process of developing relationships to and with Web3 in ways that align with their specific cultural, geographic, linguistic, cultural, and legal realities. In understanding potential value or harm of Web3, this note encourages IPs to start by first determining how they will relate to it in their ways of being. Fundamentally, this note hopes to add to the growing chorus of international IPs indigenizing Web3 so that IPs might leverage these technologies for the wellbeing of Indigenous peoples, lands, waters, cultures, languages, and ways of being.

In Section 1, this note will review some Web2 data frameworks, including Indigenous Data, Big Data, and Open Data. Section 2 examines some of the constituent parts of Web3, including cryptocurrency, smart contracts, decentralized oracles, non-fungible tokens (NFTs), decentralized apps (dapps), decentralized finance (DeFi), Decentralized Autonomous Organizations (DAOs), and blockchain supply chain. Section 3 considers the UN Declaration on the Rights of Indigenous Peoples and works to construct an international Indigenous human rights framework for IPs to draw on when incorporating Web3 into IDSov. Section 4 confronts the early disturbing, dystopian exertions of neocolonial Web3 domination.

This note concludes that Web3 and the Metaverse will have significant impacts on IDSov, likely positive *and* negative. Web3 might strengthen IDSov for IPs that exert their sovereignty and begin mastering the decentralized building blocks. However, Web3 also poses some worrying threats for IDSov. Indeed, the threats to IDSov are precisely why this note concludes Web3 and the Metaverse are the upcoming battlegrounds for IDSov.

## 2. Laying a data foundation

Before diving into Web3, this note will begin by orienting its discussion and focusing first on data in general. Data is a valuable economic and cultural resource that is used in increasingly meaningful ways to shape and tailor people's virtual worlds. Indeed, many have likened data to oil in the current information extraction era. Data is harvested in many disturbing and unethical ways and then used and analyzed—often without the knowledge or valid consent of the party from which it was extracted. Incredibly sophisticated social control programs have been developed for military applications and unleashed on the world to influence elections (Democracy Now!, 2020). However, data isn't merely confined to the current age of surveillance information capitalism. Indeed, Indigenous peoples have been sophisticated data analysts' data since time immemorial (Deloria, 1999).

### 2.1. Indigenous data

A basic understanding of data can be summed up as a unit of measurement or record that generates knowledge through conceptual thinking. The western view sees data as facts derived from measurement or analysis that generally must be processed to be interpretable. In the current digital age of technology and research, more people might assume that data should be limited to computable numbers. However, Indigenous people and Indigenous researchers have recognized that data is derived in various formats. Historically, some of the first mechanisms of data recording have come from art found in cave paintings, pottery, totem poles of the Pacific Northwest. Even dances, songs, oral teachings, and specimen samples have been ways of preserving and generating knowledge. While data comes in many forms, this note focuses mainly on virtual data.

IPs have maintained complex data systems since time immemorial and are now exerting sovereignty over virtual data. As noted in the groundbreaking work *Indigenous Data Sovereignty: Toward an Agenda*:

Indigenous data sovereignty thus refers to the proper locus of authority over the management of data about Indigenous peoples, their territories and ways of life. Early expressions of Indigenous data sovereignty can be seen in Indigenous oral traditions, which included a complex set of rights and response (Taylor and Kukutai, 2016).

In June 2018, the United States Indigenous Data Sovereignty Network (USIDSN) convened in Australia for an Indigenous Data Sovereignty Summit and adopted the following definition: “Indigenous Data Sovereignty is a global movement concerned with the right of Indigenous peoples to govern the creation, collection, ownership and application of their data”.^1^ IDSov, though still a relatively young area, has proven its durability and led to new forms of conceptualizing Indigenous data. Connected to, but distinct from, the concept of IDSov, is Indigenous Data Governance (IDGov) (Tsosie, 2019; Carroll et al., 2020). Where IDSov focuses on asserting that Indigenous data belongs to IPs, IDGov shows how data is managed throughout the data lifecycle.

No matter the data's characterization, inherent in an Indigenous Data framework is an understanding the IPs must, as a precursor to analyzing data, conceptualize and gather data in ways that are culturally appropriate to them. To that end, many IPs place immense cultural importance on the concepts of relationships. Indeed, as Cree Scholar Shawn Wilson eloquently explains that to IPs, “an object or thing is not as important as one's relationships to it. This idea could be further expanded to say that reality is relationships or sets of relationships. Thus, there is no one definite reality but rather different sets of relationships that make up an Indigenous ontology” (Wilson, 2008).

One example of harmful exploitation of Indigenous data comes from a case of theft from the Havasupai tribe and their genetic material collected by Arizona State University (ASU) (Sterling, 2011; Tsosie, 2019). ASU collected blood samples from Havasupai people, supposedly for diabetes research purposes.^2^ ASU then used the Havasupai samples after supposedly completing the study, sharing them among other researchers, and analyzing them far beyond the Havasupai scope of consent (see text footnote 2). Beyond the unjust economic exploitation, the research itself attacked their traditions and ways of being (see text footnote 2). The Havasupai case is a perfect illustration of how the respective cultural values embedded within conceptions of data shape the decisions made. Under a non-Indigenous framework, data is often little more than something to be *used* for the purposes of profit. From this perspective, it is no surprise why researchers would have felt empowered to misuse the Havasupai data. To the Havasupai however, that data was the essence of how they related to themselves, one another, and existence.

Of course, IPs in the US are not the only ones who've grappled with the challenges presented by the prospective exploitation of their genetic data. Indeed, researchers in Aotearoa, New Zealand have found that there are three challenges between IPs and genetic research including (1) recognizing there is no single Indigenous perspective; (2) building Indigenous capacity and capability for future leadership in genomic research; and (3) recognizing and ameliorating the risks to Indigenous researchers (Caron et al., 2020). In this way, some of the key areas for IDSov are access, ownership, use and reuse, Indigenous-centered collaborations, and cultural relevance.

### 2.2. Big data

Big data is the information age's extractive resource industry, facilitating centralized entities' capitalistic and colonial expansion into the digital realm. Professor Chidi Oguamanam defines big data as “massive-volume, high-velocity and high-variety information assets on a scale beyond the capacity of conventional or isolated data processing applications, and convertible into diverse and far-reaching uses by powerfully endowed entities” (Kitchin, 2014; Oguamanam, 2020). The “powerful entities” that own, sell, and analyze big data include the FAAMG giants (Facebook, Apple, Amazon, Microsoft, and Google). These companies have developed sophisticated engagement algorithms to keep people on their devices and analyze their every click and view, which can then be sold to third-party marketers and analyzed to encourage user actions. As such, the tracking software used by FAAMG runs automatically with authorized consent from users, generally hidden in plain sight amongst their terms and conditions, which are designed to dissuade average users from reading. On the one hand, big data collection and analysis can be used to influence individuals and sway individual activity. On the other hand, big data can also effectively be employed at a larger scale against groups and subgroups.^3^

### 2.3. Open data

Open data is widely believed to be less exploitative than big data but maintains many significant connections to and similarities with big data. While big data exploits user data for profit, open data exploits user data for a more public oriented use. Oguamanam explains that open data and big data have “a nuanced relationship” because they are generally “constructive and modified forms of proprietary i.e., exclusive and commercial use of data in self-interested ways that strategically encourage target forms of sharing via licensing or related schemes to optimize value” (Oguamanam, 2018). Open data has focused primarily on opening up public data for public use—especially the data produced by state agencies and publicly funded research (Kitchin, 2014). Notably, discussions of open data widely recognize that sensitive and personal data should generally remain private. For example, while tribal governments in the US are certainly government entities, the notion of public governmental transparency takes on a different tenor when discussed in relation to IPs. Moreover, unlike the US state and federal governments, IPs occupy a different political reality that is diverse and multi-faceted.

## 3. The decentralized building blocks of Web3

Decentralized technologies are the building blocks of Web3 and the Metaverse. The technical landscape is ever shifting, and projects are constantly emerging, stalling, and falling apart. Therefore, tracing the contours of these pillars can be a challenging but rewarding endeavor for IPs. Web3 envisions a decentralized and democratized form of the internet that eschews the centralized Web2 monopolies exploiting users through proprietary protocols. Where Web2 is highly centralized and amenable to censorship, Web3 aims to be censorship resistant through its decentralization and immutability using the following technologies.

### 3.1. Cryptocurrencies

Cryptocurrencies are digital currencies that operate independently of central banks through blockchain technology. Bitcoin, widely known for being the world's first cryptocurrency, invented blockchain technology. The pseudonymous creator, Satoshi Nakamoto, explained in the *Bitcoin White Paper* that Bitcoin was a technology that allowed for decentralized, peer-to-peer, trustless digital transactions (Nakamoto, 2019). Blockchain solved the double-spend problem that had plagued affected previous iterations of digital currencies put forth by the Cypherpunks in the 1980s (Russo, 2020).

Since Bitcoin emerged in 2009, countless cryptocurrencies and related controversies have sprung forth. Beyond Bitcoin, numerous pyramid scheme-type scams have been commonly referred to as “rug pulls”. One incredible example is the Squid Games cryptocurrency “SQUID”, which rose from over $.01–2,800 in a few months. SQUID served no technical purpose and was purely based on the popular Netflix show *Squid Game*. The anonymous creator then liquidated all of their holdings, closed the website, and disappeared—tanking the coin's value (Binder, 2021). In addition to the currencies, the exchanges through which people purchase and trade the currencies have also proven problematic. Most famously, the popular crypto exchange FTX collapsed and has been mired in countless fraud and money laundering criminal investigations.^4^ Similarly, many retail investors were scammed by the countless Initial Coin Offering (ICO) projects that have failed since the investor frenzy of 2017 (Sedgwick, 2018; Rosic, 2020).

For better or worse, cryptocurrencies will likely play a key role in Web3. Moreover, IPs were quick to grasp the potential of minting sovereign currencies (Dobson, 2021). Cryptocurrencies will likely be used for purchasing through and interacting with Web3. For now, many regulatory questions remain around cryptocurrencies. In the United States, the Federal Reserve is researching Centralized Digital Bank Currencies (CBDCs) as it plans to launch what many fear to be such a currency under the moniker “FedNow” (AP NEWS, 2023; Board of Governors of the Federal Reserve System, 2023). Some commentators fear that government issued CBDCs could be used to exert political and economic control that would have far-reaching negative economic impacts (White, 2018; Michel, 2022). As such, Indigenous usage of these technologies presents some risks.

Cryptocurrencies have the potential to directly connect individuals and IPs globally by enabling them to interact with Web3 and transact without the involvement of centralized financial intermediaries. While it remains unclear whether such removal of centralized financial intermediaries would be positive for IPs, there is a lot of evidence that suggests that the current financial structure has been decidedly negative for IPs. For example, international centralized financial institutions such as the World Bank have often benefitted from the exploitation of IPs (Anderson and Chavkin, 2015; ICIJ, 2015; Amnesty, 2021; Tzay, 2023). Beyond the blatantly exploitative investments and development strategies, IPs have also often been excluded altogether from standard centralized financing options which has left their communities without access to capital and credit (Miriam and Akee, 2017). The systemic economic exploitation of IPs has led some scholars to call on “the modern economy [to] address the myths, structures of economic exclusion, and invisibility of colonialism expressed as capitalism” by developing radically new economic systems altogether (Hilton, 2021).

### 3.2. Blockchain

Blockchain technology is the technology that underpins the now widely known cryptocurrency Bitcoin. While cryptocurrency was the first and primarily intended use for blockchain technology, there are now numerous applications that are not currency-based and may well-revolutionize data and communications technologies that have already changed countless aspects of human existence in the recent past. Bitcoin itself came out after years of work by the Cypherpunks and many other crypto-anarchist groups looking to create an uncensorable digital currency (Russo, 2020).

The three primary principles of blockchain are immutability, decentralization, and distributed consensus (Dobson, 2021; IBM; Marr, B.). These three principles combine to develop systems which proponents claim are censorship-resistance and trustless because they allow for peer to peer interaction without any form of centralized control or verification. However, these claims are not without their issues as seen with the ongoing debate over the distributed consensus model, proof of work (POW) known colloquially as “mining” (Russo, 2020). In a POW system like Bitcoin, miners use their computers in races to solve complex decryption equations to validate transactions and receive rewards. POW mining was devised to ensure network security by preventing bad actors from attacking the network through an algorithm which makes it increasingly difficult and expensive for miners to receive rewards (Gervais et al., 2016). The ever-increasing need for graphical power has somewhat paradoxically led to the centralization of mining operations in the form of corporate mining operations (Beikverdi and Song, 2015). Moreover, this system has led many to criticize Bitcoin's insatiable need for electricity (Karmakar et al., 2021; Schincku, 2021; Dongna et al., 2023). These criticisms have also pushed the second most popular blockchain network, Ethereum, to migrate from POW to a less energy intensive consensus mechanism called Proof of Stake (POS) (Vashchuk and Shuwar, 2018; Kapengut and Mizrach, 2023).

Inherent within the three pillars of blockchain is the concept of trustlessness. Under legacy systems, a centralized authority is typically required to verify and validate transactions. Blockchain systems, however, do not require such centralized control and thus are often considered “trustless” (Learn, 2023). While communal trust and reciprocity are often considered staples of Indigenous existence, moving toward a trustless future may appeal to many IPs who, in many cases, have experienced great hardships because of the countless legacies of broken promises by colonial nations.^5^ Therefore, the ability to develop uncensorable IP-led economic and ownership systems which operate on consensus and without the need for centralized verification or control might appeal to many IPs. However, IPs should also consider the environmental impacts of the systems that they use and ensure that any blockchain networks they use align with their cultural values with respect to its environmental impacts.

### 3.3. Smart contracts and decentralized oracles

Smart contracts are automatically executing contracts managed and recorded on the blockchain (Raskin, 2016; Russo, 2020). Smart contracts are programs to execute upon the conditions established in their code without needing a centralized intermediary. The first smart contract platform was Ethereum, but many smart contract-enhanced platforms are now available. They are a central element of Web3 and all blockchain-based functionality, more advanced than a simple transactional database. In essence, smart contracts can be seen “as simple if/then statements that allow people to program decentralized agreements [which are then] stored on a blockchain. However, unlike traditional contracts—which have traditionally been enforced through courts—smart contracts are automatically enforced by the decentralized network upon completing the programmed conditions” (Dobson, 2021).

Bridging the gap between blockchains and off-chain data will be critically important in Web3. Decentralized oracles are sophisticated, smart contracts that connect blockchains with off-chain data sources such as Internet of Things devices (smart thermometers, grid edge technologies, etc.) and one another. Moreover, harmonizing blockchain-enabled smart contracts with real-world data creates space for developing new innovative kinds of smart contract deployment options. Of particular note are micro-insurance plans, which offer decentralized, automated, claimless insurance by drawing on big and open data to automate payments when certain verifiable conditions are met (e.g., crop insurance could cover rain above X inches, etc.) (Licorish, 2021).

### 3.4. Non-fungible tokens

Non-Fungible tokens (NFTs) have received much media attention lately but remain misunderstood in the mainstream. Many unbelievable and confusing headlines have abounded about purchasing strange-looking digital art pieces called NFTs. As of the time of writing, Bored Ape Yacht Club #6924, CryptoPunk #7804, and CryptoPunk#7523 are among the most expensive NFTs in history.

One of the most expensive NFT purchases ever was *Everydays: The First 5,000 Days*, which sold at a famous auction house Christie's, for $69.3 Million (Malwa, 2021). Vignesh “Metakovan” Sundaresan purchased it reportedly to “show Indians and people of color that they, too, could be patrons, that crypto was an equalizing power between the West and the Rest, and that the global south was rising” (Malwa, 2021). However, it's worth noting that some commentators have cast aspersions on the legitimacy of Sundaresan's purchase; noting that Sundaresan recouped millions after making his purchase by selling fractional ownership interests of the piece through a crypto-based investment firm (Maxwell, 2021).

Even mainstream players like Visa have started engaging in the NFT marketby purchasing a “Crypto Punk” for the equivalent of $150,000 USD (Feuer, 2021). On Opensea.io, the largest NFT marketplace, the Cryptopunks collection has been the most expensive NFT collection and has seen trading volumes of 750,018.31 ETH (OpenSea, 2023). These incredible prices that NFTs have garnered have led many to believe, likely with some justice, that the prices of NFTs were being artificially inflated by money laundering and, thus that the technology is doomed (McCall, 2021). While NFTs remain largely unregulated, there is evidence that this is beginning to change. In the United States, for example, the Internal Revenue Service released a notice of its intent to issue guidelines for the treatment of certain NFTs as collectibles for tax purposes.^6^

However, the utility of NFTs far exceeds the mere sale of strange-looking digital art; the NFT art craze has largely obscured NFTs. NFTs are more than digital art. Rather, NFTs are the record of ownership for a digital asset, which could be anything. Other NFT applications include digital collectibles, as evidenced by the development of NFTs by non-crypto businesses such as professional football clubs like Paris Saint Germain (Kuchefski, 2021). Another fascinating potential area for NFTs is the e-gaming space, as evidenced by projects like (CryptoKitties, 2023), Axie Infinity,^7^ and Gods Unchained.^8^

Unsurprisingly, IPs have been quick and pragmatic in their assessment of the potential value that NFTs could bring to their communities. Indeed, NFTs have quickly emerged as a promising avenue for Indigenous artists. Anishinaabe artist, Quinn Hopkins, made and sold a 3Dbear NFT based on a traditional design and sold it for 0.5 Eth in 2021 (Compton, 2021). Through photos and videos, powerful virtual spaces for culture have also leveraged NFT technology for exhibits to document Native American fashion, culture, and sacred lands (Collaborative Harris Sisters, 2021). There are now also virtual galleries that sell native art NFTs to international customers and other innovative cultural and environmental protection projects (Crypto, 2022; Luo, 2022; Crypto Native Art Collective, 2023).

Other possible Indigenous NFT use cases include the indigenous minting and issuance of NFT-based digital certificates of authenticity for artist members, who then could be included in sales to verify the authenticity of an item. IPs could establish sovereign virtual marketplaces to sell member-made crafts, which then come with a indigenous issued NFT that guarantees the authenticity of the item sold (Sundararajan, 2022). Similarly, it might be possible for IPs to pair the emerging NFT technologies with the growing body of work on Traditional Knowledge Labels (“TK Labels”) and the principals of OCAP^®^.^9^ Beyond the possible uses for NFTs within an IDSov framework, NFTs have broad applicability in Web3 through a myriad of potential applications where tracking the ownership or authenticity of a digital asset is paramount (Rean, 2022).

However, NFTs also present IPs with a growing area of concern around cultural appropriation. Māori scholar, Dr. Karaitiana Taiuru, observed that “[i]n the short time that Non Fungible Tokens have been created, traded and advertised online there is a large amount of cultural appropriation and stolen images being transformed into…offensive [NFT]'s being portrayed as authentic Māori culture” (Karaitiana, 2022). Similarly, Māori photographer Rawhitiroa Bosch has voiced concern about the appropriation of Māori imagery available online into NFTs by non-Māori people (Hurihanganui, 2022). To make matters worse, blockchain elements of immutability and uncensorability make enforcement actions against culturally appropriative NFTs present novel IDSov challenges. Therefore, while there are innovative and positive examples of IPs successfully leveraging NFTs, there are also negative examples of non-IPs unjustly profiting from culturally insensitive and appropriative NFTs.

### 3.5. Decentralized applications and finance

Decentralized Finance (DeFi) is a form of financial technology (“fintech”) built on blockchain and distributed ledger technologies. This has been one of the fastest evolving sectors of Web3, likely for both the opportunities and risks that it presents. DeFi seeks to innovate on traditional centralized financial services by offering trustless, and still largely unregulated, financial services such as access to currency exchances, credit, insurance, derivatives (Blockchain Wharton and Digital Asset Project, 2021). As the Wharton School at the University of Pennsylvania notes, “DeFi is a general term for decentralized applications (Dapps) providing financial services on a blockchain settlement layer, including payments, lending, trading, investments, insurance, and asset management. DeFi services typically operate without centralized intermediaries or institutions and use open protocols that allow services to be programmatically combined in flexible ways” (Blockchain Wharton and Digital Asset Project, 2021). For IPs, DeFi might present an opportunity to escape the harsh economic realities imposed on them by centralized colonial governments and exploitative centralized financial intermediaries. However, any hopes of economic freedom should be tempered considerably for the risks and concerns noted below.

According to Ethereum, a Dapp “is an application built on a decentralized network that combines a smart contract and a frontend user interface. On Ethereum, smart contracts are accessible and transparent – like open APIs – so your dapp can even include a smart contract that someone else has written”. Dapps will be a backbone of Web3, which aims to leverage decentralization to democratize the internet, make it censorship-resistant, and end the current practice of data harvesting and sales. Dapps could be used in numerous indigenous use cases such as peer-to-peer energy trading, rent and utilities payment services, cultural resources, and even language learning dapps. With respect to DeFi, Dapps are the user-facing applications that connect the smart contracts and oracles powering the financial services.

Unsurprisingly, DeFi has attracted considerable regulatory attention within the Web3 ecosystem, and likely for good reason.^10^ In its 2022 report on DeFi, the Federal Reserve Board report “discusses a generic set of stability issues that arise from the provision of financial services on blockchains, and it also highlights some unique concerns arising from the development of DeFi, especially the governance of the code used in dapps”.^11^ The detailed report ultimately concludes that “[t]he provision of financial services on public, permissionless blockchains has come a long way since the creation of Bitcoin, but DeFi has not yet reached the point of becoming systemically important. Nevertheless, the rapid growth in the role of such blockchains suggests that policymakers should start considering a full range of financial stability issues that could arise should such activities become systemically important”.

Professor Saule Omarova's incisive research notes that “[f]intech may present a unique opportunity to correct the increasingly problematic imbalance between private misallocation of credit and the public's ability to modulate credit aggregates, or it may further intensify that imbalance…[but] argues that the more established fintech applications to date are already exhibiting signs of skewing the balance further in favor of private actors' unrestrained freedom to generate—and over-generate—financial risk” (Omarova, 2019). Professor Omarova's concerns are echoed powerfully by the US Department of Treasury concluded its April 2023 report, *Illicit Finance Risk Assessment of Decentralized Finance* (Department of Treasury, 2023). The Treasury Department notes that “criminals use DeFi services to profit from illicit activity, in particular ransomware, theft, scams, drug trafficking, and proliferation finance” (Department of Treasury, 2023). Despite this concern however, the Treasury Department also notes reasonably “that illicit activity is a subset of overall activity within the DeFi space and, at present, the DeFi space remains a minor portion of the overall virtual asset ecosystem. Moreover, money laundering, proliferation financing, and terrorist financing most commonly occur using fiat currency or other traditional assets as opposed to virtual assets” (Department of Treasury, 2023).

In sum, while there are some exciting potential applications for IPs to leverage Dapps and DeFi, there are also significant causes for concern. While many centralized financial systems have often been built atop Indigenous lands and labor, a new decentralized face doesn't necessarily mean that these new systems will have a positive impact on IPs. These causes for concern provide additional support for the argument that IPs should work to conceptualize and regulate these technologies in accordance with their particular cultural and regulatory systems. As the World Economic Forum concludes in its 2021 *DeFi Policy-Maker Toolkit*, “[e]ven when there are no clear answers, policymakers are best served by considering the right questions to ask, appreciating the points of interaction and tension with their regulatory regimes, and estimating the costs and benefits of various courses of action” (World Economic Forum, 2021).

### 3.6. Decentralized autonomous organizations

Decentralized Autonomous Organizations (DAOs) are a new trustless form of self-government that relies on smart contracts and decentralized technologies for organizational decision-making. DAOs could significantly impact the leadership and administration of Indigenous governments, economic development entities, housing entities, etc. For example, a DAO could enable IPs to use their phones or other web-enabled devices to vote, come to collective decisions, and record those decisions in the blockchain (Kodzilla, 2021; Ethereum.org, 2022). Similarly, DAOs are often a preferred decentralized governance choice for DeFi services where the token holders themselves exercise voting power to make the executive decisions for the service (World Economic Forum, 2021). IPs might be able to develop immutable and verifiable blockchain voting, allowing for more direct involvement of IPs, especially those with widespread diaspora populations. These decisions, however, also come with a certain amount of risk. Researchers at MIT have forcefully rejected voting on blockchain and argued that these systems might pose problems without proper capacity, security, or oversight (Park et al., 2021).

### 3.7. Blockchain supply chains

Blockchain technology can be used to verify the authenticity of a physical item in the supply chain. By tagging an item, a box, a crate, etc., with an RFID chip connected to the blockchain, you could develop trustless systems that automatically track the movement of goods, thereby verifying authenticity. As the items move through the physical supply chain, their movement will be tracked and recorded on the blockchain through RFID, QR Codes, or Near-Field Communication (NFC). Blockchain-optimized supply chain technology could benefit IPs with businesses that maintain large supply chains. Additionally, verifying the authenticity of Indigenous-produced goods—arts, crafts, foods, medicines, etc.—could help protect the value of authentic Indigenous goods over the fakes.

Similarly, blockchain technology might enable IPs to better protect and manage their lands. IPs could preserve sacred sites and natural resources like rivers, forests, sacred sites, fishing sites, etc., by erecting blockchain scanning sites which could be used to privilege access to cultural sites, fishing grounds, etc. IPs could then develop blockchain-connected access cards that could permission access to the areas by acting as blockchain-secured digital access keys. Blockchain supply chains also present an interesting solution for managing sustainable fisheries—especially in international waters—which could synergize with Indigenous fishing rights.^12^

## 4. International human rights law, Web3, and IPs

### 4.1. UN Declaration on the Rights of Indigenous Peoples and Web3

The UN Declaration on the Rights of Indigenous Peoples (UNDRIP) provides significant confirmation for the Indigenous inherent rights over Web3, the Metaverse, and their constituent decentralized building blocks. Moreover, Web3 presents IPs with unique economic, social, political, and cultural development opportunities. Therefore, UNDRIP protects a strong basis for Indigenous assertions to productive use and sovereign control over Web3.

#### 4.1.1. Inherent rights and self-determination

Indigenous authority springs forth from the inherent sovereignty of IPs. UNDRIP begins by recognizing a diverse set of inherent Indigenous rights in its Preamble.^13^ Beyond the recognition of inherent rights, UNDRIP also recognizes the Indigenous right to actively advance and develop those inherent rights through the principle of self-determination.^14^ Self-determination encompasses IPs being entitled to be “in control of their own destinies” (Anaya, 2009; Di Blase and Vadi, 2020). More specifically, Articles 3, 4, 23, and 32 protect the rights to self-determination as it relates to the development of political, economic, cultural, and social institutions.^15^ Therefore, exerting inherent authority over Web3 and the Metaverse fall well within UNDRIP's myriad protections of self-determination.

#### 4.1.2. Indigenous cultural futurisms

UNDRIP also broadly recognizes the rights of Indigenous Peoples to meet the future needs of their peoples. Given the increasingly digital nature of human existence, many IPs are turning to digital technologies to manifest, practice, develop, and teach their religious traditions, customs, and cultures. Articles 11 and 31 protect the Indigenous right to their technologies and an interconnected cultural past, present, and future.^16^ Article 12, taken together with Article 11, protects the physical objects and virtual sites used to store, facilitate, and disseminate spiritual, religious, customary, and ceremonial information.^17^ Similarly, Articles 13 and 25 recognize the right to uphold maintaining past and future manifestations of language and spiritual practices.^18^ Taken together, IPs have broad rights under the Declaration over any objects or systems related to their past, present, and future manifestations of culture, ceremony, and technology. Therefore, any technologies and virtual spaces used to facilitate, host, or conduct cultural and ceremonial activity should be entitled to exclusive Indigenous privacy and control under inherent sovereignty as affirmed by international indigenous human rights law.

## 5. Web3's novel risks

While Web3 might present IPs with many opportunities as explored above, it is also vitally important to recognize some of the novel risks it presents. Although Web3 is still in the relatively early stages of development as compared with Web2, there have already been negative impacts on IPs and disturbing experiments at the hands of corporations and non-governmental organizations (NGOs). This section seeks to call Indigenous attention to some of the concerning developments wherein Web3 technologies have been used by corporations, nations, and non-governmental organizations to oppress and marginalize people generally and IPs more specifically.

While Web3 will likely be a highly disruptive technology, Web2 giants have already begun their efforts to absorb its benefits while leaving behind the core tenant of decentralization. Big data companies like Meta are intent on controlling large swaths of the metaverse through the use or development of private corporate blockchains (Zickgraf, 2021). While it remains unclear how impactful such corporate projects to control Web3 will be, scholars have also explored the tensions inherent in blockchain's adoption by corporations (Hütten and Thiemann, 2018).

A growing number of countries have also begun various national blockchain experiments ranging from external investment and economic development projects to full autocratic control of their citizens. Some scholars have argued that the immutable and unstoppable nature of blockchains makes them “a-legal” and thus beyond the reach of standard legal protections when fundamental rights are violated on blockchains (Schrepel, 2019). However, these arguments haven't stopped El Salvador or China from working to benefit from blockchains in ways that might have negative impacts on their citizens. In El Salvador, the government has encouraged citizens to set up crypto wallets on their phones by offering $30 USD worth of Bitcoin to everyone that does so (Hart, 2022). While touting the value of Bitcoin for remittances and banking the unbanked, the President, Nayib Bukele, also sees Bitcoin as a way to bring in external development (Roy, 2021). However, significant concern has been expressed about the potential impacts of such Bitcoin-based economic development on democracy in the country (Fadel et al., 2021; Howson, 2021a; Sinclair, 2021). Moreover, it remains unclear whether Bukele's gambles on Bitcoin will pay off financially (Rosen, 2023). Indeed, even if El Salvador's gambling ultimately proves profitable for the country as a whole, it seems that its experiment has failed and proven to be little more than a substitution of traditional centralized capital for decentralized digital capital which has ultimately had little meaningful positive impact on the people of El Salvador.

Where El Salvador might hope to stimulate external economic development, China has taken the opposite approach. In 2019, China announced its digital RMB currency, called the Digital Currency Electronic Payment (DCEP) (Vincent, 2020). In 2020, China announced its blockchain initiative called the Blockchain-based Services Network (Sung, 2020). In September 2021, China banned all cryptocurrency transactions, citing the growing risk to its financial system and facilitation of financial crime (Shin, 2022). The World Economic Forum has since speculated, likely with some accuracy, that China sought to prevent capital flight (Shin, 2022). In February 2023, China announced its national blockchain cluster which has been dubbed the “Honeycomb” (Hale, 2023). The network can reportedly handle 240 million smart contract transactions per second and is intended to underpin the transactions across a number of industries including “online shopping, hospital registrations, financial settlements between enterprises, government collaboration, and much more” (Hale, 2023).

If blockchain endeavors by nation states aren't disturbing enough, even humanitarian NGOs have begun showing interest. In 2017, the World Food Programme (WFP) launched its BuildingBlocks initiative (Raul et al., 2018; Perlitt, 2022). Through this program, the WFP scanned the retinas of Syrian refugees and uploaded them onto the Ethereum blockchain (Zambrano et al., 2018). The eye scans were connected to Ethereum wallets funded by donations which could then be used by refugees to purchase food at participating stores (Zambrano et al., 2018). While the WFP reportedly phased out the iris scanning, the experiment itself remains deeply disturbing (Zambrano et al., 2018).

Turning to IPs more specifically, blockchain technologies have already been used directly to colonize indigenous lands. Blockchain technology has also been used to colonize Fijian lands at the hands of the Asian Development Bank (ABD) (Howson, 2021b, 2022; Ottenhof, 2021). In Fiji, the ADB has developed a blockchain-based land registry project in an effort to “harmonize” government records so as to incorporate Indigenous lands into the global market and bring in predatory land purchasers.^19^ Fijian IPs resisted colonial efforts under the British Empire to take their lands through a land-claims commission process. Dr. Olivier Jutel, a Professor at the University of Otago, has said “that the proposed blockchain registry would destroy that resistance technique while prepping the land for investment from the global market”.

## 6. Conclusion

We are in the midst of a silent data revolution that will have a significant global impact and IDSov. Web3 is still in its early stages, and its constituent parts remain fraught with numerous important technological, social, and environmental questions. Indeed, the relative youth of Web3 presents opportunities for IPs in that many colonial nations have not yet enacted comprehensive regulations. However, despite the relative youth of this technology, it already presents incredible threats to IPs and their data sovereignty. Despite Satoshi Nakamoto's purportedly optimistic intentions, many might wish for a world without blockchain.

In tackling this question, this note encourages IPs to begin to conceive of, relate to, and regulate Web3 in manners consistent with their cultural values. While there are no singular ways in which IPs can benefit from or be harmed by Web3, we encourage IPs to begin the process of understanding and connecting with these technologies. Some IPs might find that these systems do not align with their cultural values and might seek to disavow these systems altogether. Others might see the utility of these systems and begin to experiment with the technologies by incorporating them into their day-to-day lives and operations. In any case, we encourage IPs to exert their inherent sovereignty, as affirmed by international human rights law, alongside or before their respective nation-states. As corporations and nation-states begin to grapple with the technologies and forces of decentralization, we believe that IPs should work to secure their IDSov by first examining and then applying and deploying these technologies in ways that align with their values to meet their needs.

Practically speaking, this might mean passing IP governmental ordinances or laws with respect to Web3 that aim to protect IP rights as the world begins to shift from centralized to decentralized data systems. Beyond announcing their rights, IPS might also benefit from the economically from these technologies by beginning to use incorporate these systems into their political economies. Whatever the particular uses ultimately look like among each IP, these uses will provide support for domestic and international legal arguments that IPs have inherent rights over Web3. This means that IPs should benefit from the positive aspects of the technology while remaining empowered to protect themselves from the negative aspects.

Where colonial blockchain efforts might serve to harm IDSov, it is critical that IPs be well-versed in these technologies so that they might defend their data and interests. For better or worse, Web3 and the Metaverse will radically change the future of IDSov moving forward. Therefore, IPs should work to position themselves to weather the brewing decentralized data storm by familiarizing with and asserting themselves over these technologies in ways that align with their respective realities. Thus, IPs must begin the process of addressing the value, whether positive or negative, of Web3 and the developing technology industries. By asserting their inherent rights in this space they may ensure that a future in a new Western digital system reflects their own interests and respects their individual ways of knowing. Through the application of their individual practices of self-governance, traditional knowledge systems, tribal codes, and cultural ways of being can potentially ensure protection against a western colonial agenda.

## Author contributions

DD drafted the initial manuscript based on work developed by all the DD. DD and AF finalized the manuscript. Both authors contributed to review and edits.

## Footnotes

^1^US Indigenous Data Sovereignty Network | Hosted by the Native Nations Institute at the University of Arizona. Available online at: https://perma.cc/3W4H-C5CR; Rainie et al. (2017).

^2^Sterling (2011); see also Havasupai Tribe of Havasupai Rsrv. v. Arizona Bd. of Regents, 220 Ariz. 214, 217, 204 P.3d 1063, 1066 (Ct. Carapella et al., 2022).

^3^Democracy Now! The Weaponization of Data: Cambridge Analytica, Information Warfare & the 2016 Election of Trump; Fuller (2019).

^4^FTX Founder Sam Bankman-Fried Pleads Not Guilty to Fraud & Money Laundering Charges. | Democracy Now! Available online at: https://perma.cc/2GUP-NNNL; Egan (2022).

^5^International Survival (2023) and Morin (2023); “Broken Promises: Indigenous Peoples' Mental Health in South America”; “Trail of Broken Treaties 20-Point Position Paper - An Indian Manifesto”. Available online at: https://www.aimovement.org/ggc/trailofbrokentreaties.html (accessed April 12, 2023).

^6^Internal Revenue Service. Treatment of certain nonfungible tokens as collectibles.” Notice 2023-27. https://www.irs.gov/pub/irs-drop/n-23-27.pdf.

^7^Axie Infinity. https://axieinfinity.com/ (accessed February 6, 2023).

^8^Gods Unchained TCG. https://godsunchained.com (ccessed February 6, 2023).

^9^TK Labels – Local Contexts. https://localcontexts.org/labels/traditional-knowledge-labels/ (Accessed April 13, 2023.); Traditional Knowledge Labels | Sq'éwlets - A Stó:Lo-Coast Salish Community in the Fraser River Valley. http://digitalsqewlets.ca/traditional-knowledge_connaissances_traditionnelles-eng.php (Accessed April 13, 2023); The First Nations Information Governance Center. The First Nations Principles of OCAP^®^. https://fnigc.ca/ocap-training/ (Accessed April 13, 2023).

^10^Carapella et al. (2022).

^11^Carapella et al., “Decentralized Finance (DeFi): Transformative Potential & Associated Risks.”

^12^Blaha and Katafono (2020); Fishcoin (2023).

^13^UN General Assembly. United Nations Declaration on the Rights of Indigenous Peoples. Adopted by the General Assembly, October 2, 2007. A/RES/61/295. Available online at: https://www.refworld.org/docid/471355a82.html (accessed February 6, 2023).

^14^UN General Assembly. “United Nations Declaration on the Rights of Indigenous Peoples.” Preamble paragraph 16, Articles 3, 4, 23, 32, & 33.

^15^UN General Assembly, “United Nations Declaration on the Rights of Indigenous Peoples.”

^16^UN General Assembly, “United Nations Declaration on the Rights of Indigenous Peoples,” Articles 11 & 31.

^17^UN General Assembly, “United Nations Declaration on the Rights of Indigenous Peoples,” Article 12.

^18^UN General Assembly, “United Nations Declaration on the Rights of Indigenous Peoples,” Articles 13 & 25.

^19^Ottenhof. Crypto-Colonialists Use the Most Vulnerable People in the World as Guinea Pigs.
